# Precision Medicine in High-Grade Serous Ovarian Cancer: Targeted Therapies and the Challenge of Chemoresistance

**DOI:** 10.3390/ijms26062545

**Published:** 2025-03-12

**Authors:** Sara Polajžer, Katarina Černe

**Affiliations:** Institute of Pharmacology and Experimental Toxicology, Faculty of Medicine, University of Ljubljana, Korytkova 2, SI-1000 Ljubljana, Slovenia; sara.polajzer@mf.uni-lj.si

**Keywords:** high-grade serous ovarian cancer, chemoresistance, ovarian cancer, targeted therapies, predictive biomarker

## Abstract

The poor prognosis for high-grade serous ovarian cancer (HGSOC), the dominant subtype of ovarian cancer, reflects its aggressive nature, late diagnosis, and the highest mortality rate among all gynaecologic cancers. Apart from late diagnosis, the main reason for the poor prognosis and its unsuccessful treatment is primarily the emergence of chemoresistance to carboplatin. Although there is a good response to primary treatment, the disease recurs in 80% of cases, at which point it is largely resistant to carboplatin. The introduction of novel targeted therapies in the second decade of the 21st century has begun to transform the treatment of HGSOC, although their impact on overall survival remains unsatisfactory. Targeting the specific pathways known to be abnormally activated in HGSOC is especially difficult due to the molecular diversity of its subtypes. Moreover, a range of molecular changes are associated with acquired chemoresistance, e.g., reversion of *BRCA1* and *BRCA2* germline alleles. In this review, we examine the advantages and disadvantages of approved targeted therapies, including bevacizumab, PARP inhibitors (PARPis), and treatments targeting cells with neurotrophic tyrosine receptor kinase (NTRK), B-rapidly accelerated fibrosarcoma (*BRAF*), and rearranged during transfection (*RET*) gene alterations, as well as antibody–drug conjugates. Additionally, we explore promising new targets under investigation in ongoing clinical trials, such as immune checkpoint inhibitors, anti-angiogenic agents, phosphatidylinositol-3-kinase (PI3K) inhibitors, Wee1 kinase inhibitors, and ataxia telangiectasia and Rad3-related protein (ATR) inhibitors for platinum-resistant disease. Despite the development of new targeted therapies, carboplatin remains the fundamental medicine in HGSOC therapy. The correct choice of treatment strategy for better survival of patients with advanced HGSOC should therefore include a prediction of patients’ risks of developing chemoresistance to platinum-based chemotherapy. Moreover, effective targeted therapy requires the selection of patients who are likely to derive clinical benefit while minimizing potential adverse effects, underscoring the essence of precision medicine.

## 1. Introduction

**Figure 1:**
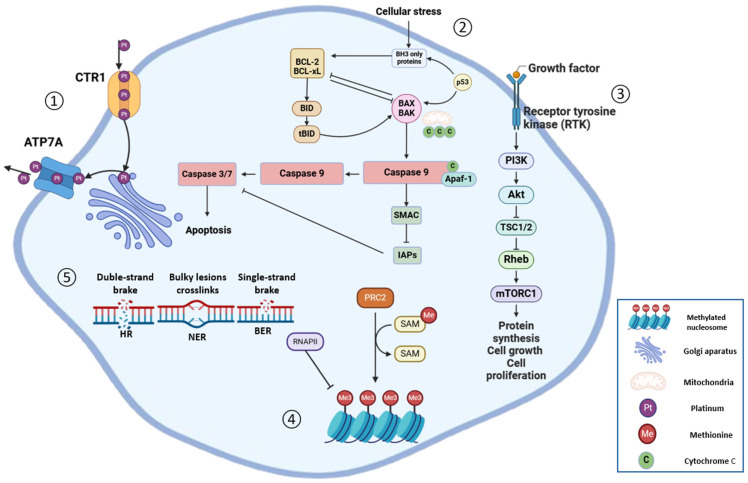
Mechanisms of platinum resistance in ovarian cancer. (1) Drug transport mechanism: Changes in the expression or function of cellular transporters, like CTR1, can reduce the uptake of platinum drugs into cancer cells, while overexpression of efflux transporters, such as ATP7A transporters, can pump these drugs out, both leading to decreased intracellular concentrations and reduced cytotoxic effects. (2) Apoptosis evasion: Cancer cells can evade apoptosis through various mechanisms, such as upregulation of anti-apoptotic proteins (e.g., Bcl-2, Bcl-xL) or downregulation of pro-apoptotic proteins (e.g., Bax, Bak). This allows cells to survive despite platinum-induced DNA damage. (3) Signalling pathways: Dysregulation of signalling pathways such as the PI3K/AKT/mTOR pathway can promote cell survival and proliferation, contributing to resistance. Activation of these pathways can also enhance DNA repair and inhibit apoptosis. (4) Epigenetic changes: Epigenetic modifications can lead to the silencing of genes involved in DNA repair, apoptosis, and drug transport. (5) DNA repair mechanisms: Upregulation of nucleotide excision repair proteins and restoration of homologous recombination repair function, through changes in genes like *BRCA1* and *BRCA2*, enable cancer cells to survive and resist platinum-induced DNA damage. Created with BioRender.com (accessed on 12 July 2024). ATP7A: ATPase copper transporting alpha; CTR1: high-affinity copper uptake protein; BCL-2: B-cell lymphoma-2 protein; BCL-xL: B-cell lymphoma-extra large protein; BID: BH3 interacting-domain death agonist; tBID: truncated BID; BAX: BCL-2-associated X protein; BAK: BCL-2 homologous killer; p53: tumour antigen p53; Apaf-1: apoptotic peptidase activating factor 1; SMAC: diablo homolog, mitochondrial; IAPs: inhibitor of apoptosis proteins; PI3K: phosphoinositide 3-kinase; Akt: protein kinase B; TSC1/2: tuberous sclerosis protein 1 and 2; Rheb: Ras homolog, mTORC binding protein; mTORC1: mammalian target of rapamycin complex 1; PRC2: polycomb repressive complex 2; SAM: S-adenosyl methionine; RNAPII: RNA polymerase II; Me: methionine; HR: homologous recombination; NER: nucleotide excision repair; BER: base excision repair.

Ovarian cancer (OC), in spite of its infrequent incidence, is the most lethal malignancy of the female reproductive system [1]. According to the GLOBOCAN database (Global Cancer Observatory established by IARC, WHO, Lyon, France), a total of 324,603 new cases and 206,956 new deaths were recorded globally in 2022 [2]. The poor prognosis of OC is primarily due to its late detection, with over two-thirds of patients diagnosed at an advanced stage, when treatment is less effective, largely because of intrinsic or, more often, acquired development of chemoresistance through mechanisms such as genetic mutations, drug efflux via transporters, enhanced DNA repair, altered cell death pathways, metabolic adaptations, epigenetic changes, and tumour microenvironment (TME) influences (Figure 1) [3,4]. Consequently, the 5-year survival rate for those that are diagnosed with OC in an advanced stage is only 29%, compared with 93% for women that are diagnosed in an early stage [5,6]. Although there has been an overall decreasing trend of incidence and mortality of OC over the past decade, a substantial increase in incidence has been observed in younger females [7]. Among other factors, the increasing incidence may also be related to the rising presence of a *BRCA* gene mutation in younger females [8], which shows that the DNA repair mechanism is an important target in pharmacotherapy for OC.

An important aspect of precision medicine is identifying novel drug targets that offer higher success rates for selected patients than existing treatments. Since every drug targets specific molecules or processes, “targeted therapy” requires precise targeting of biologically relevant processes in cancer cells, distinguishing them from normal cells. The target must be measurable in the clinic, with its measurement correlating with clinical outcomes [9]. OC is not a single disease but a group of malignancies with various targets, all presenting in the same anatomical location. Our focus is on high-grade serous ovarian cancer (HGSOC), the most common and lethal form of epithelial ovarian cancer (EOC), which accounts for over 90% of OCs [5,10]. The standard treatment for HGSOC has remained largely unchanged for decades, involving cytoreductive surgery followed by either postoperative (adjuvant) chemotherapy or chemotherapy before surgery (neoadjuvant) [11]. However, the introduction of targeted therapies in the 2010s has improved progression-free survival (PFS) compared to chemotherapy alone. Still, these therapies have highlighted a key issue: HGSOC exhibits considerable molecular diversity. Tumour heterogeneity, which may be temporal or spatial, complicates treatment [11,12] Consequently, targeted therapy for OC presents two challenges: (1) incomplete understanding of response determination, and (2) limitations in current tests used to identify eligible patients for targeted therapies [5,9,10,11,12,13,14]. Resistance to chemotherapy or targeted drugs remains a major clinical obstacle, limiting the success of HGSOC treatments.

Here, we review the pros and cons of already-approved targeted therapies. In addition, we present promising new targets included in ongoing clinical trials. Furthermore, we discuss the crucial importance of establishing robust predictive biomarkers that we can reliably measure in the clinic and thereby ensure full success of precision medicine for HGSOC.

## 2. Approved Targeted Therapies—Pros and Cons

### 2.1. First-Line and Maintenance Therapies

There are two classes of pathway-targeted OC medicines: monoclonal antibodies (bevacizumab) that recognize the cell surface and shed antigens, and small-molecule inhibitors (olaparib, niraparib, rucapari, larotrectinib, entrectinib) that can enter cells and engage intracellular targets. A monoclonal antibody is generally specific for a single antigen; it has a long half-life and requires only intermittent parenteral administration. Small molecules often inhibit multiple enzymes with different selectivities and are thus likely to have a broad spectrum of targets, and produce a broader spectrum of desired effects, off-target effects, and adverse effects than monoclonal antibodies. Many small molecules have an elimination half-life of 12–24 h and typically require at least daily oral administration [13]. Currently, all approved targeted therapies are indicated for advanced-stage disease, recurrent cancer, or patients with no satisfactory treatment options (Table 1). For patients with stage I–II HGSOC, the current recommendation remains adjuvant platinum-based chemotherapy [15,16].

#### 2.1.1. Poly (Adenosine Diphosphate-Ribose) Polymerase Inhibitors (PARPis)

PARPis are oral small-molecule inhibitors of PARP enzymes that have recently demonstrated great clinical efficacy in advanced HGSOC patients, especially in terms of significantly improved progression-free survival. Currently, three PARPis (olaparib, niraparib and rucaparib) have been approved by the European Medicines Agency (EMA) [34,35,36] and Food and Drug Administration (FDA) [37,38,39] for the treatment of HGSOC (Table 2). Inhibition of PARP enzymes, especially PARP1 and, to a lesser extent, PARP2, is an example of how a greater understanding of the underlying mechanisms of disease can lead to targeted, more personalized, treatment.

**Figure 2:**
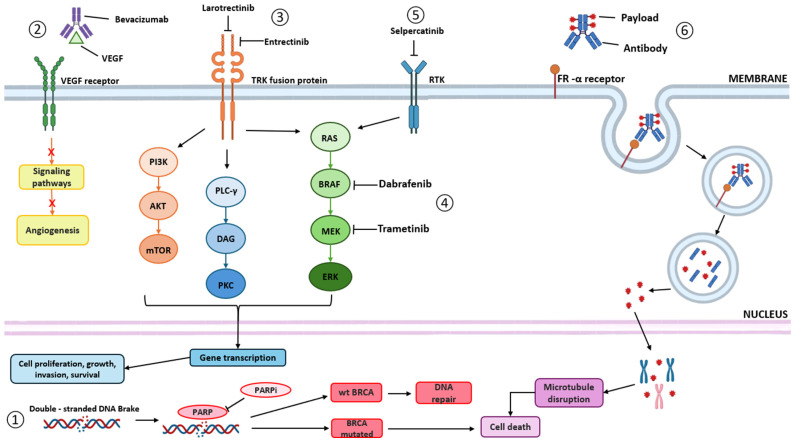
Molecular targets for targeted therapy: The figure shows targets and mechanisms of action of PARPis (1), bevacizumab (2), TRK inhibitors larotrectinib and entrectinib (3), *BRAF* inhibitor dabrafenib and MEK inhibitor trametinib (4), *RET* inhibitor selpercatinib (5), and antibody–drug conjugates (6). PARP: poly (ADP-ribose) polymerase protein; PARPi: PARP inhibitors; BRCA: breast cancer protein; VEGF: vascular endothelial growth factor; TRK: tyrosine kinase; PI3K: phosphoinositide 3-kinase; AKT: protein kinase B; mTOR: mammalian target of rapamycin; PLC-γ: phospholipase C gamma; DAG: diacylglycerol; PKC: protein kinase C; RAS: rat sarcoma virus; BRAF: B-Raf proto-oncogene, serine/threonine kinase; MEK: mitogen-activated protein kinase kinase; ERK: extracellular signal-regulated kinase; RTK: receptor tyrosine kinase; FR-α: folate receptor alpha.

PARPs are a family of enzymes that have an important role in several cellular processes, including chromatin remodelling, stress response, DNA repair, cell proliferation and cell death. PARPs have the ability to trigger auto-PARylation in the presence of DNA strand breaks, as well as to catalyse the transfer of ADP-ribose to target proteins (poly ADP-ribosylation) [40,41]. The first and the most well characterized member of the PARP family is PARP1, identified for its role in the repair of single-strand breaks (SSBs) using the base excision repair (BER) pathway [42] and nucleotide excision repair (NER) pathway [41]. DNA breaks are detected through the conserved N-terminal DNA-damage sensing and binding domain of PARP, resulting in activation of PARP1 and leading to the enzymatic cleavage of nicotinamide adenine dinucleotide (NAD+), which generates nicotinamide and ADP-ribose. Subsequently, multiple ADP-ribose units are successfully added, creating long chains of poly (ADP-ribose) (PAR). This auto-PARylation further activates PARP1 and enables the PARylation of histones and other DNA repair proteins. They form extensive polymers in close proximity to the sites of DNA damage. These polymers carry a high negative charge and serve as a structural scaffold, attracting and recruiting various critical proteins involved in BER and single-strand break repairs (SSBRs), such as XRCC1 [40,42] (Figure 2). PARPis aim to exploit HRD, particularly in BRCA-mutant tumours, to enhance the cytotoxic effects of platinum-based chemotherapy and potentially boost immune responses by increasing neoantigen presentation. These agents inhibit DNA repair mechanisms, leading to synthetic lethality in HR-deficient cancer cells, while also modulating immune pathways like STING and PD-L1, which may improve the efficacy of immune checkpoint inhibitors (ICIs) such as pembrolizumab [43,44].

**Figure 3:**
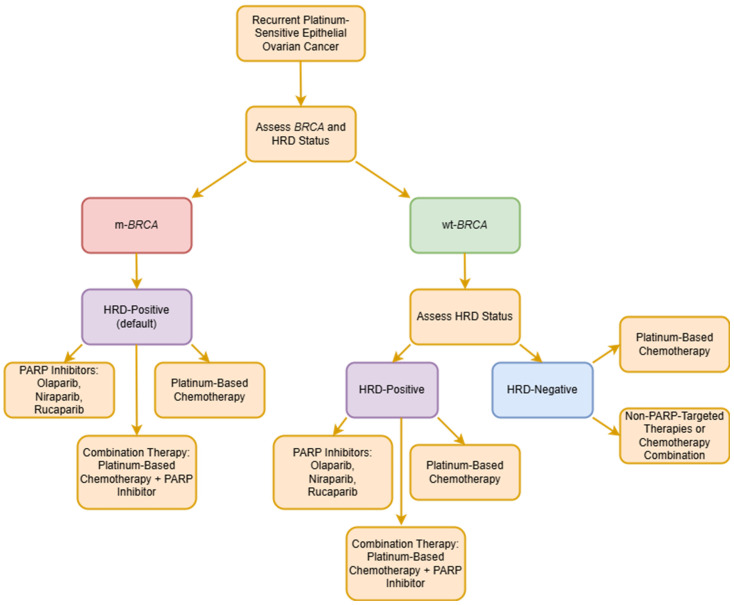
Algorithm for the treatment of patients with recurrent platinum-sensitive, epithelial ovarian cancer based on BRCA and HRD status.

PARPis are indicated as monotherapy for maintenance treatment in the first-line and relapsed settings of adult patients following response (complete or partial) to platinum-based chemotherapy [45]. Thus, one of the disadvantages of PARPis is that they are not an option for platinum-resistant disease. Based on the results of clinical trials, clinicians have various options for using different PARPis based on the presence/absence of molecular markers. In newly diagnosed HGSOC, olaparib is indicated in *BRCA1/2*-mutated HGSOC, olaparib plus bevacizumab in patients with homologous recombination deficiency (HRD), and niraparib independent of HRD or *BRCA* status. For recurrent platinum-sensitive ovarian cancer, olaparib is available for *BRCA*-mutated patients, while niraparib and rucaparib are approved by the EMA regardless of HRD or BRCA status. However, the FDA restricts niraparib to patients with germline *BRCA* mutation [45,46] (Table 1) (Figure 3). With these options, we are now able to delay the progression of HGSOC and thereby the point at which the patients will require subsequent therapy. This indirectly slows down the development of acquired resistance to platinum-based chemotherapy. However, mature overall survival (OS) data from first-line studies are not available yet. Data from a SOLO-2 trial on maintenance olaparib in relapsed HGSOC with *BRCA* mutation showed an improvement of 12.9 months in median overall survival vs. a placebo, although a statistical difference was not reached [47].

There has not to date been a randomized controlled trial that directly compares the efficacy and safety of different PARPis. Moreover, clinical trials that have tested different PARPis differ in many ways (e.g., differences in inclusion criteria and the control groups), so comparisons between them are problematic. A recent meta-analysis of six clinical trials observed no statistical difference in efficacy among olaparib, niraparib, and rucaparib in terms of improved progression-free survival (PFS) over a placebo. The results implied that olaparib had the fewest grade 3-or-higher adverse events [48].

From a pharmacological point of view, PARPis are different in certain characteristics. Rucaparib has a wider spectrum of inhibition of PARP enzymes, beyond PARP1 and PARP2. In addition, off-target kinase activity has been described for niraparib and rucaparib. In therapeutic doses, both inhibit important kinases, such as DYRK1s, VD16, and PIM3. PARPis also differ in their pharmacokinetic properties. Niraparib is not metabolized by hepatic cytochrome and has less potential for drug–drug pharmacokinetic interactions. The co-administration of olaparib with strong or moderate CYP3 inhibitors/inducers is not recommended. The absorption of olaparib is influenced by food, so co-administration with food should be avoided [49,50].

The safety profiles of PARPis share some similarities, although there are also differences among them, so an individual PARPi can be chosen according to the patient’s characteristics. Contraindications, hypersensitivity to the active substance or to any of the excipients and breast-feeding, are identical for all three PARPis. Haematologic adverse effects (anaemia, thrombocytopenia, neutropenia) are very common in PARPi therapy, with anaemia as the most common grade 3-or-worse treatment-related adverse effect. Thrombocytopenia and neutropenia are additional very common grade 3-or-worse adverse effects in treatment with niraparib. Thrombocytopenia seems to be associated with a reversible inhibition of megakaryocyte proliferation and maturation [51]. Due to haematologic adverse effects, dose adjustment or a dose interruption period (maximum of 28 days) of niraparib is necessary in patients with a platelet count < 100,000/µL, neutrophil count < 1000/µL, or haemoglobin < 8 g/dL. Additionally, reduction of the niraparib starting dose for patients less than 58 kg may be considered.

In relation to gastrointestinal adverse effects, very common adverse effects of all three PARPis are nausea, vomiting, diarrhoea and dyspepsia. Constipation is additionally a very common adverse effect in treatment with niraparib. Among gastrointestinal disorders, vomiting and nausea are common grade 3-or-worse treatment-related adverse effects, while diarrhoea is a common serious adverse effect only in treatment with olaparib and rucaparib. Both nausea and vomiting are intermittent for the majority of patients and can be managed by dose interruption, dose reduction, and/or antiemetic therapy. In the final analysis of a SOLO-2 trial, intestinal obstruction was reported among the most common serious treatment-emergent adverse effects of olaparib [47]. In addition to the already mentioned, the most frequently (≥10%) observed adverse effects of all three PARPis are decreased appetite, dizziness, dyspnoea, and fatigue, among which fatigue is the only common grade 3-or-worse treatment-related adverse effect of PARPis. In addition to thrombocytopenia and neutropenia related to olaparib [52], the most relevant differences in serious adverse effects among the three PARPis are psychiatric disorders (insomnia, anxiety, depression, confusional state), hypertension, hypokalaemia, pneumonitis, urinary tract infections, bronchitis related to treatment with niraparib [51], and increased blood creatinine, hypercholesterolaemia, and increased serum aminotransferases related to treatment with rucaparib [53].

With long-term outcome data now available, it is evident that the primary safety concern associated with PARPis is their link to an increased risk of myelodysplastic syndrome (MDSL) or acute myeloid leukaemia (AML). Data from clinical trials differ between the recurrent and first-line settings, with reported rates of MDS or AML ranging from 0.5–1.5% for PARPi use in the first-line setting to as high as 8% in the recurrent setting [45,47]. Real-world data further confirm the actual risk of developing myeloid neoplasms in OC patients following chemotherapy and prolonged PARPi therapy. The risk of myeloid neoplasms is multifaceted. Among other factors, PARPis may (a) cause bone marrow suppression, leading to persistent cytopenia; (b) exert selective pressure on hematopoietic stem cells, promoting the expansion of TP53-, DNMT3A-, and TET2-mutated pre-leukemic clones; and (c) impair DNA repair in hematopoietic stem cells, allowing the accumulation of pre-leukemic mutations. Molecular analysis revealed that 68% of secondary leukemias in PARP-treated patients harboured missense mutations in the DNA-binding domain of TP53. Additionally, a higher number of total carboplatin cycles and low platelet counts during PARPi therapy were associated with an increased risk of secondary leukemias [13,45]. Furthermore, solid tumours, including breast, endometrial, and pancreatic cancers, have been reported in long-term survivors on PARPis. However, a clear causal relationship has not been well established.

In addition to secondary cancers, long-term effects of PARP inhibitors also include fatigue, which may persist for over two years, as well as cardiovascular and neurological adverse effects [54].

**Table 2:** Current drug approval status: Food and Drug Administration (FDA) and European Medicines Agency (EMA) [13,47].

Drug Name	FDA Approved	EMA Approved	References
**Olaparib**	✓	✓	[20,34]
**Niraparib**	✓	✓	[22,35]
**Rucaparib**	✓	✓	[24,36]
**Bevacizumab**	✓	✓	[18,55]
**Larotrectinib**	✓	✓	[25,26]
**Entrectinib**	✓	✓	[28,56]
**Dabrafenib + Trametinib**	✓	✕	[31]
**Selpercatinib**	✓	✓	[57,58]
**Mirvetuximab Soravtansine**	✓	✓	[32,33]

Approved: ✓; Not approved: ✕.

#### 2.1.2. Bevacizumab

Bevacizumab is a humanized monoclonal antibody directed against vascular endothelial growth factor (VEGF). Bevacizumab binds to VEGF and thereby prevents the binding of VEGF to its receptors, Flt-1 (VEGFR-1) and KDR (VEGFR-2), on the surface of endothelial cells (Figure 2). Neutralizing the biological activity of VEGF regresses the vascularization of tumours and inhibits the formation of a new tumour vasculature and thereby inhibits tumour growth and metastasis. Interestingly, the delay of tumour growth induced by the anti-VEGF antibody has been mainly attributed to the blockage of ascites development and vascular permeability and to a lesser degree to the inhibition of VEGF-induced angiogenesis [59,60]. Because it targets a process that is essential for the growth of a solid tumour, it is no surprise that bevacizumab has a special place in therapy; it has been approved by the EMA and FDA (Table 2) for (1) primary therapy in combination with platinum-based chemotherapy; (2) post-primary maintenance therapy as monotherapy in homologous recombination (HR)-proficient/status unknown tumours or in combination with olaparib in HRD tumours (for both options, bevacizumab should be used during primary chemotherapy); (3) recurrence therapy, in combination with chemotherapy of platinum-sensitive or platinum-resistant recurrent HGSOC (patients who have not received prior therapy with bevacizumab or other VEGF inhibitors or VEGF receptor-targeted agents) [61,62].

One of the most frequent adverse events associated with long-term treatment of HGSOC with bevacizumab and chemotherapy is hypertension. This may result from VEGF inhibition, which decreases nitric oxide production in the endothelium, leading to vasoconstriction, increased peripheral vascular resistance, and elevated blood pressure. The second most common adverse event after prolonged treatment is proteinuria, induced by VEGF inhibitors through the underexpression of glomerular nephrin and subacute glomerular thrombotic microangiopathy. These changes are characterized by endotheliosis and membranoproliferative alterations [63].

Patients with a poorer prognosis may derive most benefit from adding bevacizumab during primary platinum-based chemotherapy and as a single-agent maintenance therapy after primary therapy. Bevacizumab improves PFS and OS in the high-risk group of patients, including those with stage IV, inoperable stage III, or sub-optimally debulked (residual disease > 1 cm) stage III [62]. It has been shown that patients with an unfavourable KELIM (CA-125 elimination rate constant K) score, which has been validated in the ICON 7 and GOG 218 trials, with high-risk advanced disease, derived significant benefit from the use of bevacizumab [64]. Due to potential interference with postoperative healing, bevacizumab in neoadjuvant chemotherapy is used after interval debulking surgery (IDS). Bevacizumab should be used with caution before IDS and should be withheld from therapy for 4–6 weeks before IDS [16]. Bevacizumab also improves OS in patients with ascites, a higher median pre-treatment CA-125 level, and a poor performance score [65]. In addition, improved PFS has been observed in patients without mutations in *BRCA1/2* or a *non-BRCA* HRR gene. However, using mutation status to identify patients who may benefit from adding bevacizumab is at present inadequate, since mutation status does not significantly modify the effect of bevacizumab on PFS [16]. No clinically relevant pharmacokinetic interaction of bevacizumab on co-administered medicines has been observed. A combination of bevacizumab with platinum- or taxane-based therapies increased rates of severe neutropenia, febrile neutropenia, or infections with or without severe neutropenia. When comparing the safety of platinum-based chemotherapy and bevacizumab, the following types of adverse effects are more commonly associated with adding bevacizumab to platinum-based therapy: bleeding, hypertension, proteinuria, thromboembolic events, gastrointestinal (GI) perforation, and wound-healing complications [59,66,67]. An analysis of GI-related adverse effects identified that inflammatory bowel disease and bowel resection at primary surgery are associated with an increased risk of grade ≥ 2 perforation, fistula, necrosis, or haemorrhage [67].

Bevacizumab’s integration into cancer treatment has revolutionized therapeutic strategies, offering a significant option for advanced cancers with poor prognosis. Its role continues to evolve, with ongoing research exploring combinations with PARPis and ICIs, particularly in OC [68,69]. A PAOLA-1 trial demonstrated that combining bevacizumab with the PARPi olaparib significantly improved PFS in newly diagnosed OC, especially in patients with BRCA mutations and HRD-positive tumours [59]. By inhibiting angiogenesis, bevacizumab further impacts the tumour microenvironment, leading to shifts in cytokine and growth factor profiles, changes in immune cell infiltration, and modifications to the extracellular matrix. These alterations can create a more favourable environment for immune responses, thereby enhancing the effectiveness of immunotherapies [59,70].

### 2.2. Therapies for Persistent and Recurrent Disease

In the context of persistent or recurrent OC, patients should undergo tumour molecular analysis to identify mutations that may inform targeted therapeutic options. Specifically, testing should include BRCA1/2 mutations and HR status, since these can guide the use of PARP inhibitors. Additionally, testing for microsatellite instability (MSI), mismatch repair (MMR) status, tumour mutational burden (TMB), and other relevant markers, such as folate receptor alpha (FRα), B-rapidly accelerated fibrosarcoma (*BRAF*) gene alternation, neurotrophic tyrosine receptor kinase (NTRK) and rearranged during transfection (*RET*) gene alternation, is crucial if these were not included in previous assessments. This comprehensive approach helps tailor treatment strategies based on individual molecular profiles, although some of these mutations are rare in HGSOC [71,72,73]. On the other hand, the identification of specific mutations can significantly influence treatment options for platinum-resistant disease. It is essential that all testing is performed in a CLIA-approved facility to ensure the accuracy and reliability of results using the most recent available tumour tissue [16].

#### 2.2.1. TRK Inhibitors

*NTRK* gene fusion-positive tumours are cancers with specific genetic abnormalities involving *NTRK* genes (*NTRK1*, *NTRK2*, *NTRK3*), which encode tropomyosin receptor kinase (TRK) proteins A, B, and C [74]. These proteins are crucial for nerve cell development and function. TRK receptors activate downstream signalling pathways on binding with neurotrophins such as nerve growth factor (NGF), brain-derived neurotrophic growth factor (BDNF), and neurotrophin 3 (NT3), promoting cell growth and survival [74,75]. When *NTRK* genes fuse with other genes, they form abnormal fusion proteins that can activate pathways promoting cancer growth [75]. *NTRK* gene fusion-positive tumours can occur in a wide range of cancer types, including but not limited to certain types of solid tumours, such as certain types of lung cancer, colorectal cancer, thyroid cancer, HGSOC, and sarcomas [71,74,76,77]. Identifying *NTRK* gene fusions is crucial for cancer diagnosis and treatment, since they can be targeted with *NTRK* inhibitors. These therapies block the activity of abnormal fusion proteins, showing promising results in treating *NTRK* gene fusion-positive tumours [74,77,78,79]. Medications approved by regulatory agencies such as the FDA (Food and Drug Administration) and EMA (European Medicines Agency) that target cells with *NTRK* gene changes in various cancer types, including HGSOC, are larotrectinib (brand name: Vitrakvi) [26] and entrectinib (brand name: Rozlytrek) [28] (Table 2).

##### Larotrectinib

Larotrecitinib is an orally administered, small-molecule TRK inhibitor that targets *NTRK* gene fusion-positive tumours. In November 2018, the FDA granted approval for larotrectinib for the treatment of both adult and paediatric patients 1 month or older afflicted by solid tumours containing *NTRK* gene fusions [80,81].

Larotrectinib’s mechanism of action is marked by its high binding affinities for all three TRK receptors (TRKA, TRKB, and TRKC), a result of obstructing the ATP binding site (Figure 2). By inhibiting TRKs, it induces apoptosis and inhibits cell growth in tumours overexpressing TRKs due to gene fusions or regulatory domain deletion. Larotrectinib has demonstrated significantly greater binding affinities for all three TRK receptors, exceeding by at least 100-fold the binding affinity observed with other kinases. This selectivity is crucial in minimizing off-target effects, thereby reducing the potential for adverse events [80,81]. Larotrectinib exhibits dose-dependent pharmacokinetics when taken orally within a range of 100–400 mg, achieving steady plasma levels within three days. Across various formulations and age groups, the drug’s absorption and pharmacokinetic profiles remain consistent in both adults and paediatric patients. Significant drug interactions can arise when larotrectinib is taken together with potent CYP3A4 inhibitors such as itraconazole or inducers such as rifampicin, necessitating dose modifications when co-administered [81].

In clinical trials (LOXO-TRK-14001 (NCT02122913), SCOUT (NCT02637687), NAVIGATE (NCT02576431)), larotrectinib showed significant antitumour activity across diverse age groups and tumour types, regardless of *NTRK* gene fusion variations [78,81,82]. The trials demonstrated a high overall response rate (ORR), indicating larotrectinib’s efficacy in inducing tumour regression among *NTRK* fusion-positive tumour patients. Notably, larotrectinib treatment led to lasting responses, with many patients achieving prolonged disease control, suggesting potential extended survival benefits. This versatility positions larotrectinib as a promising therapeutic option for patients with various *NTRK* fusion-positive tumours [74,80,83].

Blocking *NTRK* pathways can lead to negative effects for a significant number of patients. These pathways play roles in pain regulation, temperature control, and hunger signals, as well as cognitive functions such as learning and memory [74]. Safety data from adult (phase I, SCOUT and NAVIGATE) and paediatric (phase I, SCOUT) trials have shown larotrectinib’s good tolerability. The majority of treatment-associated events were reported as mild to moderate (grade 1 or 2), manifesting in 88% of treated patients [81,82,84]. Severe adverse events (grade 3 or 4) are relatively rare but may include anaemia, weight gain, decreased neutrophil count, and increased levels of liver enzymes (alanine aminotransferase or aspartate aminotransferase) [74,82].

Dose reductions due to adverse events, such as elevated liver enzyme levels, dizziness, or decreased neutrophil count, have been reported in a small percentage of patients, typically resulting in the maintenance of their best treatment response at the lower dose [78]. Despite these dose adjustments, discontinuation of larotrectinib due to adverse events has been infrequent, particularly among patients who demonstrated a positive treatment response [74,80]. Neurologic adverse reactions emerged as notable concerns, with a significant proportion of patients experiencing events such as delirium, dysarthria, and dizziness. These neurologic events were prevalent, particularly within the initial months of treatment [85]. Additionally, gastrointestinal disturbances, including diarrhoea and constipation, have been reported in a subset of patients [81,82]. While larotrectinib’s safety and tolerability are generally well established, it is crucial for healthcare providers to remain vigilant for potential adverse events, particularly those affecting the liver, nervous system, and haematologic parameters. Close monitoring and prompt intervention can help optimize patient outcomes while minimizing the impact of treatment-related adverse events.

##### Entrectinib

Entrectinib, a first-generation TRK inhibitor, has gained approval for both adult and paediatric patients aged 12 and older [74,83]. It effectively targets TRKA, TRKB, and TRKC receptors, decreasing cellular proliferation and survival in cancers with *NTRK* gene fusions (Figure 2). Additionally, entrectinib inhibits ROS1 and ALK, making it beneficial for metastatic, ROS1-positive non-small-cell lung cancer (NSCLC). This orally available inhibitor demonstrates high CNS (central nervous system) concentrations, crossing the blood–brain barrier and showing promise in treating primary solid tumours, including those with brain metastases, thus addressing an unmet medical need [85,86,87].

Primarily metabolized by CYP3A4, entrectinib interacts significantly with CYP3A4 substrates (e.g., midazolam), inhibitors (e.g., itraconazole), and inducers (e.g., rifampicin). Its main active metabolite inhibits transporters MATE1, BCRP, and OATP1B1, suggesting potential drug interactions. Despite these considerations, clinical data underscore entrectinib’s efficacy across various fusion partners and tissue histologies, making it a promising therapeutic option for multiple cancers [86]. Caution is advised when co-administering with moderate and strong CYP3A4 inhibitors, necessitating dose adjustments. Mild-to-moderate renal impairment does not require dose adjustments, but severe renal impairment has not been studied. Similarly, mild hepatic impairment does not warrant adjustments, while moderate-to-severe impairment has not been studied. Additionally, dosing of entrectinib is not dependent on food intake [85].

Entrectinib has emerged as a promising therapeutic option for patients with *NTRK* fusion-positive solid tumours and ROS1-positive NSCLC, as demonstrated by findings from the ALKA-372-001, STARTRK-1, and STARTRK-2 studies. These trials collectively showed that entrectinib elicited clinically meaningful and durable systemic responses across various solid tumour types, including those with CNS metastases at baseline. The studies reported notable objective response rates and durable responses, with entrectinib exhibiting efficacy in both adult and paediatric populations. Additionally, the safety profile of entrectinib was generally favourable, with manageable adverse events reported across the studies. Overall, the findings from the ALKA-372-001, STARTRK-1, and STARTRK-2 studies underscore the clinical benefit of entrectinib in patients with *NTRK* fusion-positive tumours and ROS1-positive NSCLC, reaffirming its role as a promising targeted therapy in oncology [74,85,86,87].

Entrectinib demonstrated a manageable safety profile across the ALKA-372-001, STARTRK-1, and STARTRK-2 studies, with most adverse events reported being of mild-to-moderate severity (grades 1–2). Common grade 1–2 adverse effects included dysgeusia, diarrhoea, and weight increase, along with fatigue, dizziness, constipation, nausea, vomiting, myalgia, peripheral oedema, paraesthesia, arthralgia, increased blood creatinine, and elevated aspartate aminotransferase levels [85,87]. The most frequently reported grade 3–4 adverse events encompass anaemia, increased weight, dyspnoea, diarrhoea, increased aminotransferase and fatigue, occurring with varying frequencies among patients [74,86]. Notably, nervous system disorders emerged as a significant concern, with cognitive disorders, cerebellar ataxia, and dizziness reported as serious treatment-related events. Management of treatment-related adverse events often necessitated dose adjustments, interruptions, or even discontinuation in some cases. Furthermore, entrectinib carries additional warnings for congestive heart failure, central nervous system effects, and skeletal fractures, underscoring the importance of close monitoring during treatment. Despite these challenges, entrectinib’s adverse event profile remains manageable, with most events reversible and serious adverse events occurring relatively infrequently [64].

#### 2.2.2. RET Inhibitors

The *RET* gene, discovered through the transfection of NIH/3T3 cells with human lymphoma DNA, encodes a transmembrane receptor tyrosine kinase (RTK) crucial for cellular signalling, renal morphogenesis, neural development, and spermatogonial stem cell maintenance [88,89]. *RET* gene fusion-positive tumours result from the fusion of *RET* with another gene, producing an abnormal RET protein that promotes cancer growth [72,89]. *RET* activation in cancer occurs via chromosomal rearrangements and gain-of-function mutations, with elevated wild-type RET expression also contributing to tumourigenesis [88,89,90]. Tyrosine kinase inhibitors (TKIs) are used to treat *RET* gene fusion-positive tumours by blocking the activity of the abnormal RET protein, inhibiting cancer cell growth. These inhibitors bind to the ATP-binding pocket of the RET kinase domain, preventing phosphorylation of downstream signalling molecules [89,90]. Selpercatinib, formerly known as LOXO-292, is a highly selective RET inhibitor. It specifically targets the abnormal RET protein, blocking its activity and thereby inhibiting cancer cell growth [90]. These fusions are common in papillary thyroid, NSCLC, and OC, indicating a poor prognosis. While *RET* gene fusions are not commonly associated with HGSOC, the National Comprehensive Cancer Network (NCCN) guidelines recommend testing for *RET* gene fusions and administering therapy with selpercatinib in patients whose tumours are positive for these fusions [16,72].

##### Selpercatinib

Selpercatinib (brand name: RETEVMO™), developed by Loxo Oncology, a subsidiary of Eli Lilly and Company, is a highly selective inhibitor of the receptor tyrosine kinase RET, designed for the treatment of various solid tumours. In vitro studies have shown selpercatinib’s potent efficacy in suppressing the proliferation of diverse cancer cell lines with *RET* alterations, while in vivo models have demonstrated its ability to induce tumour regression (Figure 2). Metabolized primarily by cytochrome P450 (CYP) 3A4, selpercatinib’s pharmacokinetics are influenced by factors such as body weight and hepatic function, with limited impact of age, gender, or renal impairment. However, coadministration with certain medications, including omeprazole, itraconazole, and rifampin, can alter selpercatinib’s pharmacokinetic profile, highlighting the importance of careful consideration for potential drug interactions during treatment [88,91].

Selpercatinib received accelerated approval from the FDA on September 21, 2022, for adult patients with locally advanced or metastatic solid tumours harbouring a *RET* gene fusion, either progressed on prior systemic treatment or with no satisfactory alternative options [57] (Table 2). The approval was based on data from the LIBRETTO-001 trial, which evaluated 41 patients with *RET* fusion-positive tumours, excluding non-small-cell lung cancer and thyroid cancer. The trial demonstrated an ORR of 44%, with a median duration of response (DOR) of 24.5 months. The recommended dose of selpercatinib varies based on body weight: 120 mg orally twice daily for patients weighing less than 50 kg and 160 mg orally twice daily for those weighing 50 kg or more [92,93]. While it has shown efficacy in certain cancers, its use in OC is not a primary recommendation according to the NCCN guidelines. However, it can prove beneficial in specific situations, particularly as targeted therapy for *RET* gene fusion-positive tumours [16].

Reported treatment-emergent adverse events, affecting at least a quarter of patients, predominantly comprised oedema, diarrhoea, fatigue, dry mouth, hypertension, abdominal pain, constipation, rash, nausea, and headache [93]. Additionally, grade 3 or 4 laboratory abnormalities, including decreased lymphocytes and elevated alanine aminotransferase (ALT) and aspartate aminotransferase (AST) levels, were observed. Notable warnings in the US product labelling highlighted potential adverse events such as hepatotoxicity, interstitial lung disease/pneumonitis, and QTc interval prolongation [92,93]. Grade 3–4 laboratory abnormalities were noted in ≥ 2% of patients, including increased AST, ALT, glucose, alkaline phosphatase, and bilirubin levels, and decreased calcium and platelet levels. Serious adverse reactions were observed in 33% of selpercatinib-treated patients, with pneumonia being the most frequent. Three percent of patients experienced fatal adverse reactions, such as sepsis, cardiac arrest, and respiratory failure, each occurring in three patients. Five percent of patients required permanent discontinuation of selpercatinib therapy due to adverse reactions, including increased ALT and AST levels, sepsis, drug hypersensitivity, fatigue, and thrombocytopenia. Furthermore, dosage interruptions due to adverse reactions were necessary in 42% of selpercatinib-treated patients, involving issues such as increased ALT or AST levels, hypertension, diarrhoea, pyrexia, and QT prolongation. Dosage reductions were required in 31% of patients due to adverse reactions, including increased ALT or AST levels, QT prolongation, and fatigue [91,92]. While selpercatinib has demonstrated efficacy in treating *RET* fusion-positive tumours, vigilant monitoring and management of adverse events, particularly those related to laboratory abnormalities and serious reactions, are pivotal components of patient care during selpercatinib therapy.

#### 2.2.3. BRAF Inhibitors

The B-rapidly accelerated fibrosarcoma (*BRAF*) gene plays a crucial role in activating the mitogen-activated protein kinase (MAPK) pathway, pivotal for cellular growth and differentiation, and is frequently mutated across various cancers. Cancer development attributed to the MAPK pathway accounts for approximately 30% of all cancers, with a significant portion stemming from a B-Raf mutation. This mutation is notably prevalent in melanoma cancer, ranging from 50% to 80%, and is also observed in thyroid cancer (36% to 53%), colorectal cancer (5% to 22%), and OC (30%). *BRAF* V600E-positive tumours are cancers that carry a specific genetic mutation known as *BRAF* V600E. The V600E mutation is a specific alteration in the *BRAF* gene by which the amino acid valine (V) is replaced by glutamic acid (E) at position 600 of the protein, and it accounts for 90% of *BRAF* mutations [94,95,96]. These mutations overstimulate the MAPK signalling pathway, persistently activating the enzymatic domain [97]. In ovarian tumours, *BRAF* mutations exhibit a dichotomous distribution, rare in HGSOC but prominent in low-malignant-potential serous ovarian tumours [73,94,95]. Early detection of these mutations is critical for determining optimal treatment strategies, with *BRAF* inhibitors showing significant advancements in PFS and OS rates among cancer patients [96,97]. These targeted therapies, designed to counteract the aberrant activity of mutated BRAF proteins, have yielded significant benefits in certain cancer types, including in HGSOC [73,97,98]. Dabrafenib, an ATP-competitive inhibitor for *BRAF* kinase, was developed by GlaxoSmithKline (GSK) and approved by the FDA for treating *BRAF* V600E/K-mutated metastatic melanoma. Despite its initial success, resistance to *BRAF* inhibitors such as dabrafenib has emerged because of various factors, such as loss of PTEN, NF1, or USP28-FBW7, CCND1 amplification, COT overexpression, and mutations in RAC1 [97]. To combat this resistance, combination therapies, such as dabrafenib plus trametinib, have been explored in clinical trials. These combination therapies target multiple signalling pathways involved in cancer growth. Dabrafenib and trametinib, inhibiting BRAF and MEK proteins, respectively (Figure 2), have shown durable responses and manageable toxicity in treating not only melanoma but also other *BRAF*-positive solid tumours, such as NSCLC, anaplastic thyroid cancer (ATC), and various other cancers [99,100,101]. Dabrafenib in combination with trametinib received accelerated approval from the FDA on 22 June 2022. This approval was granted for the treatment of unresectable or metastatic solid tumours with a *BRAF* V600E mutation in both adult and paediatric patients aged six years and older who have progressed after prior treatment and lack satisfactory alternative treatment options [31] (Table 2). The approval was based on trials involving 131 adult patients and 36 paediatric patients, showing ORRs of 41% and 25%, respectively. Common adverse reactions in adults included pyrexia, fatigue, nausea, and rash, while paediatric patients experienced pyrexia, rash, vomiting, and fatigue. The recommended doses vary based on age and weight, with dabrafenib at 150 mg orally twice daily and trametinib at 2 mg orally once daily in adults [100,101]. According to the NCCN guidelines, the combination of dabrafenib and trametinib is not the standard treatment for OC. However, it can prove beneficial in specific situations, particularly as targeted therapy for OC carrying the *BRAF* V600E mutation [16]. Reliable detection of the V600E mutation is of critical importance in HGSOC, offering prospects for personalized treatment options and improved therapeutic outcomes in these patients [73].

#### 2.2.4. Antibody–Drug Conjugates (ADCs)

ADCs are a sophisticated class of targeted cancer therapies combining the specificity of monoclonal antibodies with potent cytotoxic agents. Comprising three essential components—the antibody, linker, and payload—ADCs are meticulously designed to deliver cytotoxic payloads specifically to tumour cells, while minimizing off-target effects [102,103]. The monoclonal antibody, typically a humanized or fully human IgG, targets tumour-specific antigens such as FRα or mesothelin [104]. On binding, the ADC–antigen complex undergoes internalization, allowing the payload to be released inside cancer cells [103,105]. Central to ADC design is the linker, a critical chemical structure that connects the antibody to the cytotoxic payload [104,106]. Cleavable linkers, sensitive to intracellular conditions, ensure the payload is released within the tumour cells, minimizing damage to healthy tissues [103]. Alternatively, non-cleavable linkers release the payload on lysosomal degradation of the antibody, offering enhanced plasma stability but lacking the bystander effect of cleavable linkers, which can affect neighbouring tumour cells [107]. Payloads such as monomethyl auristatin E (MMAE) and maytansinoid (DM4) are chosen for their potent cytotoxic effects at low concentrations, disrupting microtubules or inducing DNA damage in rapidly dividing tumour cells [104].

##### Mirvetuximab Soravtansine

Mirvetuximab soravtansine is an ADC designed to target FRα, which is preferentially overexpressed on malignant epithelial cells, particularly in OC and endometrial cancers (Figure 2). The ADC consists of a humanized FRα-binding monoclonal IgG1 antibody (M9346A) linked via a cleavable disulfide linker to the cytotoxic maytansinoid DM4, which inhibits tubulin polymerization with greater potency than vinca alkaloids [107,108]. This design allows mirvetuximab soravtansine to target FRα-positive cells effectively while also generating lipophilic catabolites capable of killing neighbouring FRα-negative cells through a bystander effect [104]. Folate metabolism, crucial for DNA, RNA, and protein methylation, as well as DNA synthesis and repair, involves high-affinity binding to FRα, making this receptor an attractive target for ADC-based therapies [102]. Platinum-based chemotherapy has been the cornerstone treatment for OC, but resistance necessitates alternative strategies [15,109]. With approximately 80% of EOC tumours expressing FRα, mirvetuximab soravtansine is a promising therapeutic approach [109]. It binds FRα and delivers a cytotoxic maytansinoid DM4 payload, demonstrating synergy with chemotherapy, particularly in platinum-resistant HGSOC. Additionally, it may induce immunogenic cell death through monocyte activation, upregulation of immunogenic markers like HMGB1 and calreticulin, and direct induction of apoptosis via its cytotoxic payload, DM4, which disrupts microtubule function. This process not only enhances the local immune response by activating monocytes and promoting dendritic cell maturation—crucial for initiating immune responses—but also creates a synergistic effect when combined with ICIs. Mirvetuximab soravtansine activates antigen-presenting cells through interactions with Fcγ receptors, increasing the presentation of tumour antigens, which promotes T-cell activation and strengthens the immune response against tumour cells. Additionally, the apoptosis induced by mirvetuximab soravtansine releases pro-inflammatory cytokines and damage-associated molecular patterns (DAMPs), which further stimulate immune activation and help create a more immunogenic tumour microenvironment, potentially improving treatment outcomes in patients with HGSOC [110,111]. Clinical trials have shown that mirvetuximab soravtansine, the first FRα-targeting ADC, exhibits significant anticancer activity and a tolerable safety profile in patients with platinum-resistant OC [112,113,114].

On 14 November 2022, the US FDA granted accelerated approval to mirvetuximab soravtansine for treating adults with FRα-positive, platinum-resistant EOC, fallopian tube, and primary peritoneal cancer, specifically for patients who have received 1–3 prior systemic treatment regimens (Table 2). This approval was based on the impressive tumour response rate and durability of response seen in the phase III SORAYA trial [102]. Patients eligible for this treatment are selected based on FRα expression, determined using an FDA-approved companion diagnostic test. Mirvetuximab soravatansine is administered as an intravenous infusion every three weeks until disease progression or unacceptable toxicity occurs [108]. Premedication with corticosteroids, antihistamines, antipyretics, and antiemetics is recommended to reduce the frequency and severity of infusion-related reactions, nausea, and vomiting [102].

In the pivotal phase III SORAYA trial, mirvetuximab soravtansine showed significant antitumour activity in patients with high FRα expression who had received 1–3 prior lines of therapy. The trial reported an ORR of 32.4%, with 4.8% of patients achieving a complete response and 27.6% a partial response. Additionally, 71% of patients experienced tumour reduction. The median duration of response was 6.9 months, and median PFS was 4.3 months, with a median OS of 13.8 months [102,112]. Further studies are ongoing, including investigations into platinum-sensitive ovarian cancer as monotherapy and in combination with bevacizumab plus carboplatin [108,113,114].

Clinical trials have demonstrated the manageable tolerability profile of mirvetuximab soravtansine. In a pooled analysis of 464 patients from three clinical trials, common treatment-related adverse events included blurred vision (42%), nausea (40%), diarrhoea (33%), fatigue (31%), keratopathy (26%), and dry eye (22%) [112,113]. Most adverse events were grade 1 or 2, manageable with supportive care and dose modifications. Severe adverse events (≥grade 3) occurred in 26% of patients, with 7% discontinuing treatment due to treatment-related adverse events [112,113,114]. Notably, ocular events and peripheral neuropathy were among the adverse events of interest, with 61% and 36% of patients experiencing these effects, respectively. Severe ocular events were rare, and most resolved to mild levels or completely with appropriate management [102,108]. Mirvetuximab soravtansine is a promising therapeutic option for patients with FRα-positive, platinum-resistant HGSOC ovarian cancer, offering a targeted approach with a manageable safety profile. Ongoing studies and confirmatory trials will further elucidate its long-term efficacy and safety across various patient populations and cancer types [73,115].

## 3. Promising Innovative Therapies

### 3.1. ICIs in Combination with PARPis and Anti-Angiogenic Agents

While traditional treatments such as surgery and chemotherapy remain standard, recent advances in understanding the molecular biology of HGSOC have led to the development of targeted therapies [116] (Table 3). PARPis have demonstrated impressive clinical activity in OC patients. However, their effectiveness as monotherapies is often limited by intrinsic and acquired resistance [117]. PARPis are being investigated in combination with other drugs, such as ICIs and anti-angiogenic agents, to enhance their efficacy [68,116]. Anti-angiogenic agents can synergize with PARP inhibitors in treating ovarian cancer by decreasing the expression of homologous recombination repair (HRR) genes, such as *BRCA1* and *RAD51*, under hypoxic conditions. The combination of olaparib and the VEGFR3 inhibitor cediranib yielded better outcomes in *BRCA*-wild-type patients with platinum-sensitive recurrent ovarian cancer compared to olaparib alone. This improvement may be attributed to cediranib’s potential to downregulate *BRCA1* and *BRCA2*, and induce HRD [69,118]. Immunotherapies have revolutionized the treatment of many solid tumours and have a strong rationale for use in HGSOC. Novel treatments such as PD-1/PD-L1 immune checkpoint blockade have been investigated for HGSOC [119,120,121]. Immune checkpoint blockade therapy works by targeting inhibitory pathways that tumours exploit to evade immune detection, primarily the PD-1/PD-L1 and CTLA-4 pathways. The PD-1 receptor, expressed on activated T cells, binds to its ligand PD-L1 on tumour or immune cells, leading to T-cell exhaustion and immune suppression. ICIs targeting PD-1 (e.g., pembrolizumab, nivolumab) or PD-L1 (e.g., durvalumab) block this interaction, restoring T-cell function and enhancing antitumour immunity. Similarly, CTLA-4, another inhibitory receptor on T cells, competes with the costimulatory receptor CD28 for binding to B7 molecules on antigen-presenting cells. CTLA-4 blockade prevents this suppression, promoting T-cell activation and proliferation. By disrupting these inhibitory signals, immune checkpoint blockade reactivates the immune system, enabling a stronger and more sustained attack against tumours [122]. The significance of the immune system in OC is highlighted by the finding that patients with tumour-infiltrating lymphocytes (TILs) show significantly improved five-year survival rates compared to those without TILs [69,117]. Selecting cancer patients for immunotherapy involves identifying biomarkers, such as PD-L1 expression and MSI/MMR status, which predict responses to ICIs. Testing should ideally include both primary and metastatic lesions, due to potential discordance in biomarker expression. Patient-specific factors, tumour type, and regulatory approvals also guide selection, while emerging biomarkers and combination approaches may further refine eligibility in the future [123]. Clinical trials are investigating the combination of pembrolizumab, an anti-PD1 antibody, with olaparib, a PARPi, and bevacizumab in *BRCA*-wild-type patients with platinum-sensitive recurrent OC. This combination is based on the potential synergistic effects of enhancing DNA change and inhibiting immune checkpoints to boost antitumour immunity [68,124]. Other studies have demonstrated synergistic effects for a similar triplet combination (durvalumab, olaparib, and bevacizumab) in platinum-resistant OC [68,69,125,126]. Another clinical trial demonstrated that the PARPi niraparib, in combination with pembrolizumab, shows promise and is well tolerated by patients with HGSOC, who have few treatment options, irrespective of their BRCA/homologous recombination deficiency status, biomarker status, or previous bevacizumab treatment [121,127]. Notably, patients without tumour *BRCA* mutations or with non-HRD cancers exhibited higher-than-anticipated responses compared to using either agent alone [117,128]. The ATHENA–COMBO comparison aims to evaluate the magnitude of the benefit of adding nivolumab to rucaparib monotherapy in a frontline maintenance setting, potentially providing additional therapeutic options to further extend PFS [129].

While pembrolizumab has not yet secured approval from the FDA or EMA specifically for OC, it is featured in the NCCN guidelines for treating solid tumours characterized by MSI-H or dMMR, as well as those with a high tumour mutational burden. Similarly, dostarlimab-gxly is recommended in these guidelines for patients with recurrent or advanced dMMR/MSI-H tumours [16]. Notably, on 17 August 2021, the FDA granted accelerated approval for dostarlimab-gxly, targeting adult patients with dMMR solid tumours that have recurred or progressed after previous therapies, particularly for those without viable alternative treatments [30]. Dostarlimab-gxly works by inhibiting the PD-1 protein found on cancer cells, which can suppress tumour activity, while also enhancing T-cell function by blocking immune checkpoints, thereby boosting the immune response against the cancer [130].

ICIs can provoke autoimmune-like reactions affecting multiple organs, sometimes emerging months or years after therapy cessation. PD-1/PD-L1 blockade lifts immune inhibition, leading to T-cell hyperactivation and potential autoimmune destruction of normal tissues (e.g., endocrine glands, lungs, liver, and GI tract). Mitigation strategies include long-term endocrine monitoring (TSH, cortisol, glucose), early detection of pneumonitis through lung imaging if respiratory symptoms arise, and the use of corticosteroids or immunosuppressants for severe immune-related adverse effects. Some studies suggest a potential risk of T-cell-driven lymphoproliferative disorders in long-term ICI users [131].

### 3.2. PI3K Inhibitors

Alpelisib (BYL719) is an α-specific phosphatidylinositol-3-kinase (PI3K) inhibitor that can be taken orally. It has 50 times the potency of other PI3K isoforms when it comes to exclusively inhibiting p110α. Alpelisib demonstrated a dual mechanism of action in preclinical trials, whereby it inhibited p-Akt and degraded p110α protein levels in a manner that was dependent on the dose. The PI3K pathway is a key oncogenic pathway that controls growth, survival, metabolism, apoptosis, and proliferation of cells. Its inhibition can cause *BRCA1* or *BRCA2* to be downregulated, HRR to be disrupted, and the cell to become more susceptible to PARPis [132,133]. The synergistic activity between alpelisib and olaparib in platinum-resistant HGSOC of the *BRCA* wild-type has been shown in an EPIK-O/ENGOT-OV16 study, thereby extending the potential use of PARPis beyond the HRR-deficient context [115,133,134].

### 3.3. WEE1 Inhibitors

Adavosertib (AZD1775) is a first-in-class, oral, reversible small-molecule inhibitor of Wee1 kinase that shows promise as an anticancer agent in advanced solid tumours, especially in cells lacking p53 [135]. Adavosertib inhibits Wee1, a regulator of the G2/M cell-cycle checkpoint, and has demonstrated efficacy as monotherapy in phase I and II clinical studies for patients with advanced solid tumours, including those with *BRCA* mutations [136,137]. The accumulation of DNA damage caused by this inhibition amplifies the cytotoxic effects. Adavosertib has shown a significant improvement in response rates, PFS, and OS in patients with platinum-resistant HGSOC when taken with DNA-damaging drugs such as carboplatin and gemcitabine [138,139,140]. Specifically, in TP53-mutated platinum-resistant OC patients, a phase II trial showed the safety and efficacy of adavosertib in combination with carboplatin [141]. Additionally, in patients with recurrent OC, combining adavosertib with a PARPi resulted in an ORR of 29%, as opposed to 23% with adavosertib alone, indicating a potential synergistic effect on improving treatment efficacy [136]. Particularly in genetically susceptible subgroups, these results encourage more research on adavosertib as a monotherapy and in combination regimens for the treatment of OC [140,142].

### 3.4. ATR Inhibitors

In the context of targeted therapy for HGSOC, several ATR (ataxia telangiectasia and Rad3-related protein) inhibitors are currently being researched. One of the key proteins in the DNA damage response (DDR) pathway is ATR kinase, which mainly serves as a replication stress sensor. Because of oncogene activation and disruption of G1 checkpoint control, replication stress is enhanced in cancer, which enables ATR to identify regions of single-stranded DNA (ssDNA) within double-stranded DNA (dsDNA) [143]. For cells to survive, DNA damage detection and repair depend on DDR pathways. Ineffective DNA repair and cell death are the results of defects in these pathways, which are prevalent in HGSOC with p53 and *BRCA1/2* mutations [144]. One new therapeutic approach that has emerged is targeting the ATR/CHK1 pathway. By transducing signals through checkpoint kinase 1 (CHK1), ATR regulates the cell cycle and can increase DNA damage and increase the lethal effects of PARPis such as olaparib by blocking HR-mediated DNA repair. The combination of olaparib and ATR inhibitors, such as ceralasertib, is promising, particularly for patients with HGSOC who have a *BRCA1/2* mutation and have not responded well to previous PARPi therapy [144,145,146].

**Table 3:** Overview of emerging therapies, their mechanisms of action, potential benefits, and examples of clinical trials.

Targeted Therapy	Mechanism of Action	Target	Potential Benefits	Clinical Trials	References
**Immune Checkpoint Inhibitors**	Block proteins that act as brakes on the immune system	CTLA-4, PD-1, and PD-L1	Enhance the ability of T cells to recognize and attack cancer cells	OPEB-01/APGOT-OV4 (NCT04361370) MEDIOLA (NCT02734004) ATHENA-COMBO (NCT03522246)	[69,124,129]
**Anti-angiogenic Agents**	Inhibit the growth of new blood vessels (angiogenesis) that supply tumours	VEGF (vascular endothelial growth factor) and its receptors	Starve the tumour of nutrients and oxygen, inhibiting its growth	CONCERTO (NCT02889900)	[5,118,147]
**PI3K Inhibitors**	Block the phosphoinositide 3-kinase (PI3K) pathway	PI3K enzymes involved in cell growth, proliferation, and survival	Reduce cancer cell proliferation and induce apoptosis	EPIK-O/ENGOT-OV16 (NCT04729387)	[132,133]
**WEE1 Inhibitors**	Inhibit WEE1 kinase, a regulator of the cell cycle	WEE1, which controls the G2/M checkpoint	Force cancer cells to enter mitosis prematurely, leading to cell death due to DNA damage	Adavosertib monotherapy (NCT02482311) Adavosertib + carboplatin (NCT01164995)	[136,137,139]
**ATR Inhibitors**	Inhibit ATR (ataxia telangiectasia and Rad3-related protein) kinase	ATR, involved in the DNA damage response	Prevent cancer cells from repairing DNA damage, leading to cell death	CAPRI (NTC03462342)	[144,145]

## 4. Predictive Biomarkers for Platinum-Resistant Tumours

**Figure 4:**
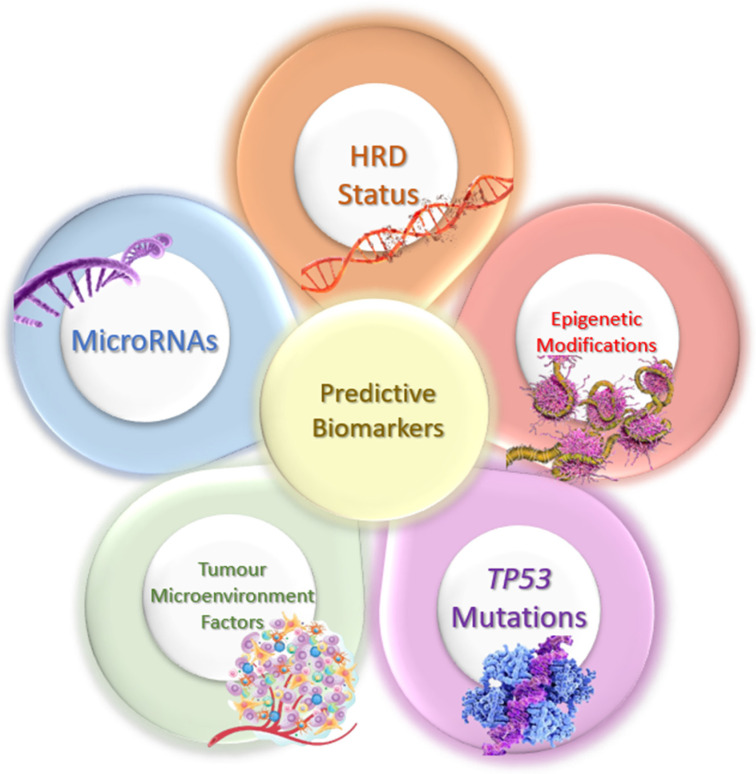
Predictive biomarkers of platinum-resistant ovarian cancer.

Biomarkers for predicting platinum resistance in HGSOC are crucial, since they play an important part in precision medicine, improve patient outcomes, and reduce unnecessary exposure to ineffective therapies (Figure 4). These biomarkers can help identify which patients are likely to benefit from platinum-based chemotherapy, allowing for the optimization of therapeutic strategies and the minimization of toxic side effects [148,149]. Establishing robust predictive biomarkers that can be reliably measured in the clinic is of great importance for several key reasons. Firstly, they enable personalized medicine, allowing clinicians to tailor treatments to individual patients based on their unique biomarker profiles. This can significantly improve therapeutic efficacy and patient outcomes by ensuring that patients receive the most appropriate and effective therapies, thus avoiding the one-size-fits-all approach that often leads to suboptimal results. Secondly, reliable predictive biomarkers can help in early identification of resistance to certain treatments, such as platinum-based chemotherapy in HGSOC [148,149,150]. By predicting resistance, clinicians can avoid administering ineffective treatments, thereby reducing the risk of serious adverse effects and preserving the patient’s quality of life [151]. This also has economic benefits, since it prevents the waste of healthcare resources on ineffective treatments and reduces overall healthcare costs [148,152].

Moreover, robust biomarkers are essential for the advancement of clinical research and the development of new therapies. They can be used to categorize patients in clinical trials, ensuring that only those likely to benefit from the particular therapy are included [151]. This increases the likelihood of trial success and accelerates the development of new targeted therapies [152].

However, achieving reliable biomarker measurement in the clinic involves overcoming several challenges. These include ensuring the reproducibility and standardization of biomarker assays, addressing biological variability among patients, and developing non-invasive or minimally invasive methods for biomarker detection. Furthermore, it requires continuous validation through large-scale clinical studies to confirm the predictive power and clinical utility of these biomarkers across diverse populations [149,150]. Additionally, the dynamic nature of cancer resistance mechanisms and the potential for biomarkers to change over time or under treatment pressure add layers of complexity to their use in clinical practice [151]. Addressing the challenges associated with reliable biomarker measurement requires a concerted effort from researchers, clinicians, and regulatory bodies to integrate these biomarkers effectively into routine clinical practice, ultimately leading to improved therapeutic efficacy, better patient outcomes, and more efficient use of healthcare resources. Several biomarkers have been identified that can predict resistance to platinum-based chemotherapy [148,152]. However, it is important to note that currently, no predictive biomarker is in clinical use specifically for predicting resistance to platinum-based chemotherapy. Among the biomarkers discussed, HRD status is primarily used for first-line maintenance therapy decisions, particularly in determining eligibility for PARPis. Epigenetic modifications, microRNAs, and TME factors have been associated with treatment response, influencing the likelihood of resistance or sensitivity to platinum-based therapies. Additionally, TP53 mutations are commonly linked to inherent platinum resistance in HGSOC [16,153].

### 4.1. HRD Status

Platinum medicines primarily target DNA, inducing cytotoxic effects by forming DNA monoadducts that can evolve into interstrand crosslinks, thereby inhibiting DNA synthesis and transcription [154]. To repair these lesions, a DNA damage response mechanism is activated, which includes a number of DNA repair pathways, such as mismatch repair, BER, NER, HRD, and nonhomologous end joining (NHEJ). Variations in these pathways can have a major impact on platinum sensitivity and resistance [154,155]. Platinum-resistant cancers frequently show upregulation of DNA repair proteins, including BRCA1/2, MSH1, MSH2, ERCC, RAD51, FANCA, and FANCG [154,156]. Tumours lacking HRD are more likely to be resistant to platinum-based chemotherapy. HRD-positive tumours, including those harbouring *BRCA1/2* mutations, generally demonstrate better responses to platinum compared to tumours with wild-type *BRCA1/2* genes. This is because *BRCA1/2* mutations impair DNA repair mechanisms, making cancer cells more susceptible to DNA-damaging agents such as platinum [155,156]. *CDK12* mutations and low expression, found in a small fraction of OC patients, are also associated with increased susceptibility to platinum and PARPis. Furthermore, changes in NER pathways, such as reduced *ERCC1* expression, can improve platinum sensitivity, although results are variable [154]. Amplification of *CCNE1* and high *ERCC1* expression have been associated with platinum resistance, while expression of *FANCA* and *FANCG* was higher in platinum-sensitive OC cell lines [154,157].

### 4.2. Epigenetic Modification

DNA methylation and histone alterations play critical roles in gene expression and have emerged as important predictive biomarkers for HGSOC. DNA methylation is the process of adding a methyl group to the fifth carbon of a cytosine ring, which is assisted by DNA methyltransferases and often happens in CpG islands found in gene promoter regions [154]. This methylation can restrict gene expression by preventing transcription factors and RNA polymerase from binding, which commonly leads to chemoresistance [157]. Studies have shown that hypermethylation of genes such as *KLF4, IL6, MSX1*, and *LAMA3* is associated with platinum resistance in OC [154,157]. Hypermethylation of the *BRCA1* promoter has also been documented in OC patients and is associated with platinum treatment sensitivity [154,157,158]. Furthermore, histone modifications, notably acetylation mediated by histone acetyltransferases (HATs) and deacetylases (HDACs), have a major impact on chromatin structure and gene expression. For example, HDAC1 has been associated with cisplatin resistance, and inhibiting it restores susceptibility to the medication. Collectively, these epigenetic changes not only promote ovarian tumourigenesis and chemoresistance, but they also provide prospective biomarkers for early detection and therapeutic targets to overcome platinum resistance in HGSOC [154].

### 4.3. MicroRNAs

MicroRNAs (miRNAs) are small, 19–25-nucleotide-long, single-stranded noncoding RNAs that play a crucial role in the post-transcriptional regulation of gene expression by binding to messenger RNAs (mRNAs) and either degrading them or inhibiting their translation. In HGSOC, multiple miRNAs exhibit altered expression patterns that are closely associated with carcinogenesis, progression, metastasis, and drug resistance, particularly to platinum-based chemotherapies [154,157,159]. Comprehensive profiling studies have identified numerous miRNAs with aberrant expression in OC cell lines and tumour tissues [154,159]. For instance, miR-10a-5p, miR-96-5p, miR-141-3p, miR-182-5p, miR-183-5p, miR-200c-3p, miR-203a, miR-296-5p, and miR-1206 have been found to be significantly upregulated in both OC samples and cisplatin-resistant HGSOC cell lines. These miRNAs contribute to platinum resistance through various mechanisms, making them potential predictive biomarkers for treatment response [159].

### 4.4. TME Factors

In HGSOC, the TME plays a critical role in influencing disease progression and treatment response, making it a valuable source for potential predictive biomarkers. Key elements of the TME, such as tumour-associated macrophages (TAMs) and immune cell infiltration, have been shown to significantly impact the clinical outcomes of HGSOC [160,161]. For instance, the presence of a high density of CD3+ T cells and CD163+ macrophages correlates with favourable responses to chemotherapy and an overall better prognosis [161]. Notably, the circITGB6/IGF2BP2/FGF9 axis has been identified as a crucial mediator in the TME, driving macrophage M2 polarization and contributing to platinum resistance. Elevated levels of circITGB6 and increased M2 macrophages in chemoresistant OC tissues further underline the importance of these factors [162]. Additionally, the ability of the TME to modulate immune responses through mechanisms such as the production of CXCL10 by M1-type TAMs, highlights the dynamic interplay between tumour cells and their microenvironment [161]. Analysing the immune contexture and specific molecular interactions within the TME can therefore provide significant predictive insights, guiding personalized therapeutic strategies and predicting chemoresistance for HGSOC patients. 

### 4.5. TP53 Mutations

The presence of specific mutations in circulating tumour DNA, such as *TP53,* can provide insights into resistance mechanisms. *TP53* mutations are notably more prevalent in HGSOC compared to other OC subtypes, and their loss of function due to inactivating mutations is associated with platinum resistance in ovarian tumour cells [163]. Treatment with platinum-based medicines has been linked to the acquisition of additional mutations in mutant *TP53* variants, such as S185G, which contribute to acquired chemoresistance [157]. Another study demonstrated that ERβ2 and mutant p53 upregulate FOXM1 expression, which is associated with increased proliferation and resistance to therapeutic agents such as carboplatin. The findings indicate that ERβ2’s role in enhancing FOXM1 transcription contributes significantly to therapeutic resistance in HGSOC [164]. The frequency of *TP53* mutations thus serves as a potential biomarker for both inherent and acquired platinum resistance, aiding in therapeutic decision making [153].

## 5. Successes and Challenges in Precision Medicine

Precision medicine in HGSOC is a significant advance, offering the promise of more effective and less toxic treatments tailored to individual genetic and molecular profiles. Success in this area has led to remarkable improvements in patient outcomes, with therapies designed specifically to attack cancer cells while sparing healthy tissue.

One major success is the use of genetic testing for *BRCA1* and *BRCA2* mutations, which has become a standard practice in determining eligibility for PARPis such as olaparib, niraparib, and rucaparib. These inhibitors have shown efficacy in prolonging PFS in patients with these mutations [165,166,167,168].

After a 7-year follow up in a SOLO1 trial, olaparib demonstrated a clinically meaningful, although not statistically significant, OS improvement as a first-line maintenance therapy for *BRCA*-mutated OC patients. Data from this trial suggest that 10-year survival appears to be an appropriate surrogate of cure [169]. For olaparib as maintenance therapy in patients with platinum-sensitive relapsed OC, Study-19 and SOLO2 reported a higher mean OS in patients with a BRCA mutation at 73% and 61% maturity, respectively. In Study-19 the difference in mean OS was statistically significant (*p* = 0.025), while in SOLO2, statistical significance was not reached (*p* = 0.054) [47,170]. Another success is the identification of HRD beyond *BRCA* mutations, broadening the scope of patients who can benefit from PARPis. Advanced testing methods and algorithms have been developed to detect HRD, thereby expanding the patient population eligible for these therapies [171]. The incorporation of bevacizumab, an anti-angiogenic agent, into treatment regimens for HGSOC patients has also been a success. By targeting the VEGF pathway, bevacizumab has improved progression-free survival, particularly in patients with advanced disease, when used in combination with chemotherapy [59]. In the PAOLA-1 trial, adding maintenance olaparib to bevacizumab delivered a clinically meaningful OS benefit for patients with HRD-positive tumours, regardless of their risk level for disease progression [172].

Furthermore, the identification of dysregulation in pathways such as PI3K/AKT/mTOR has led to the exploration and use of pathway inhibitors such as alpelisib. These inhibitors offer a targeted approach to inhibit tumour growth and potentially overcome resistance to other therapies [132]. MEK inhibitors, such as trametinib, have shown success in targeting the MAPK/ERK pathway, providing additional therapeutic options for patients with specific genetic alterations [100].

Additionally, ICIs, including pembrolizumab and nivolumab, have been identified as potential treatments for HGSOC by blocking proteins such as PD-1 or PD-L1. While their single-agent efficacy has been modest, ongoing research and clinical trials are exploring their use in combination with other therapies to enhance response rates [68,129].

Emerging research is highlighting the crucial role of microelements and their influence on ovarian cancer (OC) risk and treatment. Zinc (Zn) and copper (Cu) are essential trace elements that regulate redox homeostasis, crucial for cancer prevention and treatment. Their interplay, reflected in the Zn/Cu ratio, has increasingly been linked to OC risk, with a ratio above 6.38 offering protective effects [173]. Zinc exerts anticancer properties by supporting DNA repair, regulating the cell cycle, and reducing oxidative stress, while copper aids metabolism and antioxidant defences [173,174]. Copper efflux transporters ATP7A and ATP7B play a critical role in OC chemoresistance by regulating intracellular copper levels. Overexpression of these transporters enhances copper export, reducing the efficacy of platinum-based chemotherapy drugs like cisplatin [3]. Additionally, ATP7A expression and ceruloplasmin levels have been investigated as potential biomarkers for treatment response, with higher ATP7A expression correlating with poor chemotherapy outcomes in HGSOC [175]. Copper chelation therapy and zinc supplementation show promise in OC treatment by inhibiting angiogenesis, enhancing chemotherapy, and promoting apoptosis [173,174]. Elevated blood lead levels, particularly above 13.6 µg/L, significantly increase OC risk, especially in BRCA1 mutation carriers. Lead induces DNA damage, disrupts cell-cycle regulation, and alters gene expression. Preventive measures such as prophylactic salpingo-oophorectomy and detoxification strategies may help mitigate this risk [176]. Selenium also plays a key role in OC prevention and treatment. Studies show an inverse relationship between selenium levels and cancer risk, with patients exhibiting lower selenium concentrations as the disease progresses. Selenium supports redox homeostasis, boosts immune function, and may reduce treatment side effects. While findings on selenium’s impact remain mixed, optimizing levels could be beneficial, particularly for those with low baseline levels [177,178].

Overall, the success in identifying patients for targeted therapy in HGSOC is a testament to advances in molecular diagnostics and precision medicine, significantly improving patient outcomes and paving the way for more effective and tailored treatments [148].

However, several challenges remain in the identification and implementation of targeted therapies for HGSOC. One major hurdle is the complexity and heterogeneity of the tumours, which can vary significantly among patients, making it difficult to identify universal biomarkers. Tumours can also exhibit intra-tumoural heterogeneity, whereby different areas within the same tumour may have distinct genetic profiles, complicating the selection of appropriate targeted therapies [148]. Therefore, investigating the genetic (e.g., HRD status, BRCA1/2 mutations) and epigenetic (e.g., BRCA2 promoter methylation) variations that drive these adaptations is crucial for optimizing treatment strategies and improving outcomes in HGSOC. PARPis show strong efficacy in HRD tumours, but resistance develops through mechanisms like BRCA restoration or alterations in replication fork stability, and loss of BRCA1 promotes methylation, which can restore HR function [43,44]. In this regard, we face several challenges. Firstly, HRD assays for predicting a PARPi response, currently available in clinical practice, are only accurate for the time at which the sample is obtained. A second challenge is that, at present, none of the DNA sequencing approaches assess the presence of HR gene reversion, as a known mechanism of clinical resistance to PARPis [179]. Another challenge is the potential detriment PARPis have demonstrated in BRCA-wild-type or heavily pre-treated populations. Specifically, the NOVA and ARIEL4 trials indicated an OS decrease in BRCA-wild-type patients when niraparib and rucaparib were used in the maintenance setting for platinum-sensitive recurrence. In 2022, the FDA withdrew approval for niraparib, olaparib, and rucaparib as monotherapy for heavily pre-treated OC due to detrimental OS data in late-line settings. Maintenance therapy indications for niraparib and rucaparib were restricted to patients with BRCA mutations, though the EMA has maintained approvals for BRCA-wild-type patients in this context [45]. Beyond PARPis, the effectiveness of other targeted therapies also depends on tumour-specific genetic alterations. Platinum-based chemotherapy, such as carboplatin, is highly effective in BRCA1/2-mutated or HRD tumours, but resistance can develop through BRCA1/2 reversion mutations that restore HRR or epigenetic silencing of pro-apoptotic genes like MLH1 [43,44]. Similarly, bevacizumab, an anti-VEGF therapy, has variable efficacy depending on angiogenesis-related mutations such as alteration of FGF or PDGF signalling and hypoxia-driven epigenetic modifications [180]. Targeted agents like larotrectinib and entrectinib work well in NTRK fusion-positive tumours but are susceptible to secondary resistance mutations [181]. BRAF/MEK inhibitors (dabrafenib and trametinib) show promise in BRAF V600E-mutated HGSOC but may be ineffective if concurrent RAS or NF1 mutations are present [182]. Likewise, selpercatinib, a RET inhibitor, is effective in RET fusion-positive cases but can be impacted by epigenetic silencing of RET [183]. Another promising agent, mirvetuximab soravtansine, an ADC targeting FRα, is most effective in tumours with FOLR1 overexpression, though resistance may arise if FRα expression is lost [184].

Technical challenges also pose significant obstacles. High-quality, reproducible diagnostic tests are essential for accurately identifying patients who will benefit from targeted therapies [152]. These tests must be integrated into clinical workflows efficiently and require rigorous standardization and validation to ensure consistent results across different laboratories and settings. The cost and complexity of these diagnostic tests can also be prohibitive, limiting their accessibility and utilization [150].

Moreover, disparities in access to advanced diagnostic tools and targeted treatments, often driven by socioeconomic factors, lead to unequal patient outcomes. Patients in low-resource settings may lack access to the necessary molecular testing and advanced therapies, exacerbating healthcare inequalities. The cost of OC treatment varies significantly based on factors such as the stage of the disease, chosen treatment modalities, geographic location, and individual patient circumstances (Table 4). Addressing these disparities requires comprehensive strategies, including policy changes, to ensure equitable access to precision medicine [185].

An additional consideration is how to combine predictive biomarkers for response in order to assess the benefit of combination therapies, such as PARPis, ICIs, and inhibitors of angiogenesis [186].

Overcoming these challenges demands ongoing research to better understand tumour biology and resistance mechanisms, advancements in technology to develop more precise and affordable diagnostic tools, and collaborative efforts across the healthcare system to integrate these innovations into clinical practice. By addressing these challenges, the full potential of targeted therapies in HGSOC can be realized, ultimately leading to improved therapeutic efficacy, better patient outcomes, and more efficient use of healthcare resources.

**Table 4:** Cost analysis of genetic tests and available treatments for high-grade serous ovarian cancer (HGSOC).

Treatment	Estimated Cost per Test (US)	Estimated Cost per Test (Europe)	Notes	References
**BRCA Testing**	USD 1500–3000	EUR 1100–2500	Costs can vary based on the laboratory and specific testing methods used.	[187,188]
**Homologous Recombination Deficiency (HRD) Testing**	USD 2500–4000	EUR 1800–3000	HRD testing may include BRCA testing as part of a broader panel.	[188,189]
**Microsatellite Instability (MSI) Testing**	USD 500–2000	EUR 300–1500	MSI testing can be performed via PCR or NGS methods, influencing cost.	[190]
**Chromosomal Instability (CIN) Testing**	USD 2000–3000	EUR 1500–2500	CIN testing methods and availability may vary, affecting cost.	[191]
**Whole-Genome Sequencing (WGS)**	USD 5000–10,000	EUR 4000–8000	WGS offers extensive data but at a higher cost and longer turnaround time.	[192]
**Whole-Exome Sequencing (WES)**	USD 1500–5000	EUR 400–4000	WES is less comprehensive than WGS but more cost-effective and faster.	[192]
**Tumour Mutational Burden (TMB) Testing**	USD 1500–3000	EUR 1–2500	TMB can be assessed via targeted gene panels or broader sequencing approaches.	[193]
**Surgery**	USD 95,000	EUR 35,000	Surgical costs depend on the procedure’s complexity, hospital fees, and geographic location. In the EU, prices vary by country and healthcare system.	[194,195]
**Carboplatin**	USD 125–500	EUR 100–250	Carboplatin is a generic chemotherapy drug, making it relatively affordable. Prices depend on dosage and healthcare provider.	[189]
**Rucaparib (Rubraca)**	USD 10,000–16,000	EUR 8000–11,500	A PARPi used for maintenance therapy in recurrent ovarian cancer. Costs are based on average monthly expenses.	[196]
**Olaparib (Lynparza)**	USD 10,000–16,000	EUR 5200–13,000	Another PARPi approved for *BRCA*-mutated ovarian cancer. Monthly costs are similar to rucaparib.	[188,196]
**Niraparib (Zejula)**	USD 10,000–18,000	EUR 5500–14,800	A PARPi approved for maintenance treatment in ovarian cancer. Monthly costs are comparable to other PARPis.	[188,196]
**Bevacizumab (Avastin)**	USD 3000–13,000	EUR 4200–8400	An angiogenesis inhibitor used in combination with chemotherapy for ovarian cancer. Costs vary based on dosage and treatment duration.	[189]
**Larotrectinib (Vitrakvi)**	USD 32,800	EUR 28,000	Approved for tumours with *NTRK* gene fusions, including some ovarian cancers.	[197]
**Entrectinib (Rozlytrek)**	USD 17,050	EUR 14,500	Similar to larotrectinib, targets *NTRK* gene fusions.	[197]
**Dabrafenib (Tafinlar)**	USD 12,000–15,000	EUR 6000–7000	Targets *BRAF*-mutated cancers; used in combination with trametinib for certain ovarian cancers.	[198]
**Trametinib (Mekinist)**	USD 12,000–15,000	EUR 6000–7000	MEK inhibitor often used with dabrafenib. Monthly cost is similar to dabrafenib.	[198]
**Selpercatinib (Retevmo)**	USD 20,600	EUR 17,600	*RET* inhibitor approved for specific gene mutations; its use in ovarian cancer is under investigation.	[199]

Note: The cost of treatment varies significantly based on factors such as the stage of the disease, chosen treatment modalities, geographic location, and individual patient circumstances. Additionally, insurance coverage and reimbursement policies can significantly influence out-of-pocket expenses for patients. PARPi: Poly (adenosine diphosphate-ribose) polymerase inhibitor. NTRK: neurotrophic tyrosine receptor kinase gene fusion. RET: rearranged during transfection. BRAF: B-rapidly accelerated fibrosarcoma. MEK: mitogen-activated protein kinase kinase. PCR: polymerase chain reaction. NGS: next-generation sequencing.

## 6. Conclusions

In summary, the management of HGSOC remains a significant challenge due to its aggressive nature, late-stage diagnosis, and high recurrence rates following initial treatment. Despite the introduction of novel targeted therapies over the past decade, their impact on OS has been limited. The molecular diversity of HGSOC subtypes and the emergence of chemoresistance, particularly to carboplatin, complicate treatment strategies. While current targeted therapies, such as bevacizumab and PARPis, offer some promise, their efficacy is not universal, highlighting the need for more precise medicine. Future advances hinge on the identification and validation of robust predictive biomarkers to optimize patient selection and enhance therapeutic outcomes. Ongoing research into new targets, including ICIs and various kinase inhibitors, provides hope for more effective treatments. Continued efforts in this direction are essential to improve the prognosis and quality of life for patients with HGSOC.

## Figures and Tables

**Table 1:** Therapeutic indications for targeted therapies in high-grade serous ovarian cancer (HGSOC) approved by the European Medicines Agency (EMA) and/or the Food and by Drug Administration (FDA). Special considerations specific to FDA approvals are indicated in italic.

Medicine	Monotherapy	Combinations	Prior Response to	Special Considerations	References
			Pt-Based Therapy		
			(Complete or Partial)		
	**Primary Therapy**				
**bevacizumab**		1. carboplatin/	N/A	1. FIGO stages IIIB, IIIC, IV.	[17,18]
		paclitaxel			
	**Maintenance therapy following completion of primary Pt-based chemotherapy (CT)**				
**bevacizumab**	yes	N/A	not specified	1. FIGO stages IIIB, IIIC, IV.	[17,18]
**olaparib**	yes	N/A	yes	1. *BRCA1/2* mutation.	[19,20]
				2. FIGO stages III, IV.	
**olaparib**	N/A	bevacizumab	yes	1. HRD-positive status.	[18,19]
				2. FIGO stages III, IV.	
				3. Following primary Pt-based CT in combination with bevacizumab.	
**niraparib**	yes	N/A	yes	1. FIGO stages III, IV.	[21,22]
**rucaparib**	yes	N/A	yes	1. FIGO stages III, IV.	[23,24]
				*FDA: not approved.*	
	**Therapy of recurrent cancer**				
**bevacizumab**	N/A	1. carboplatin/	yes	1. First recurrence.	[17,18]
		gemcitabin		2. No prior anti-VEGF therapy.	
		2. carboplatin/			
		paclitaxel			
**bevacizumab**	N/A	1. paclitaxel	no	1. No more than 2 prior CT regimens.	[17,18]
		2. topotecan		2. No prior anti-VEGF therapy.	
		3. doxorubicin PL			
	**Maintenance treatment of recurrent cancer**				
**bevacizumab**	yes	N/A	not specified	Not specified	[17,18]
**olaparib**	yes	N/A	yes	1. *BRCA1/2*-mutation.	[19,20]
**niraparib**	yes	N/A	yes	Not specified	[21,22]
				*FDA: germline BRCA-mutation.*	
**rucaparib**	yes		yes	Not specified	[23,24]
	**Patients who have no satisfactory treatment options**				
**larotrectinib**	yes	N/A	no	1. *NTRK* gene fusion.	[25,26]
**entrectinib**	yes	N/A	no	1. *NTRK* gene fusion.	[27,28]
				2. No prior *NTRK* inhibitor therapy.	
**selpercatinib**	yes	N/A	no	1. *RET* fusion.	[29,30]
**dabrafenib +**	N/A	N/A	no	1. *BRAF V600E* mutation.	[31]
**trametinib**				*FDA only approved.*	
	**Pt-resistant tumour**				
**mirvetuximab**	yes	N/A	no	1. FRα positive.	[32,33]
**sorvatansine**				2. One to three prior systemic treatment regimens.	

CT: chemotherapy; PL: pegylated liposomal. HRD: homologous recombinant deficiency (defined by either BRCA1/2 mutation and/or genomic instability). NTRK: neurotrophic tyrosine receptor kinase gene fusion. RET: rearranged during transfection. BRAF: B-rapidly accelerated fibrosarcoma. N/A: not applicable.

## Data Availability

Not applicable.
